# Online education and the mental health of faculty during the COVID-19 pandemic in Japan

**DOI:** 10.1038/s41598-022-12841-x

**Published:** 2022-05-30

**Authors:** Yosuke Kita, Shoko Yasuda, Claudia Gherghel

**Affiliations:** 1 Mori Arinori Institute for Higher Education and Global Mobility, Hitotsubashi University, 2-1, Naka, Kunitachi, Tokyo, 186-8601 Japan; 2 Cognitive Brain Research Unit (CBRU), Faculty of Medicine, University of Helsinki, Helsinki, Finland; 3 Department of Psychology, Faculty of Letters, Keio University, Tokyo, Japan

**Keywords:** Psychology, Quality of life

## Abstract

While the negative impact of the pandemic on students’ mental health has been studied around the world, very little is known about the mental health of faculty and staff. This research aims to examine mental health among Japanese faculty members who taught online courses during the COVID-19 pandemic. We recruited 537 university faculty members and assessed their mental health using the World Health Organization-Five Well-Being Index (WHO-5), both retrospectively (during the academic year before the onset of the pandemic) and during the pandemic. We also evaluated workload (number of online lectures taught and preparation time per class), difficulty in using information technology (IT) for online classes, and satisfaction with the university support service for online education. As a result, the WHO-5 score during the COVID-19 pandemic was significantly lower than before, and 33.5% of the faculty members were recognized as being at risk for mental illness during the COVID-19 pandemic. A binomial logistic regression analysis revealed two significant risk factors for mental illness—faculty members were more at risk for mental illness when they experienced difficulty in using IT for online classes, and were unsatisfied with the administrative support for online education. The deterioration of mental health during the COVID-19 was not predicted by workload, such as the number of online lectures and preparation time. These results suggest the importance of improving workplace support services, especially IT support, to prevent mental health deterioration among faculty teaching online.

## Introduction

Efforts to stop the spread of the COVID-19 pandemic, such as lockdowns and the practice of physical distancing, have severely affected the quality of life of the general population. Shut downs brought about not only a financial crisis, but also social isolation and the need to rapidly adapt to major lifestyle changes. Recent evidence has revealed that stress, anxiety, depression, and poor sleep are common psychological reactions to the COVID-19 pandemic around the world^1–3^. In Japan, psychological distress increased from the early stages of the outbreak in January 2020 to the community transmission phase in April 2020^4^. Furthermore, during the national state of emergency (April–May 2020), the mental health of the Japanese population worsened compared to previous years, the most adversely affected being healthcare workers, young people, and those with a history of treatment for mental illness^5^. The deterioration of mental health is of particular concern amid a recent increase in the number of suicides among Japanese women^6^ and young people^7^. Considering that the pandemic continues to affect various aspects of individuals’ everyday functioning, preventive measures and early interventions are called for^8^.

Higher education has also been affected by the spread of COVID-19. Most university campuses around the world are closed, or have shifted from face-to-face to online education. Campus closures and social isolation have disrupted students’ learning, and caused them significant psychological distress. Many students found the emergency online education difficult to cope with, and said they preferred face-to-face instruction^9^. These changes contributed to a decrease in students’ psychological well-being. Research shows that mood disorders, stress, alcohol consumption^10^, depression, and anxiety^3,11,12^ increased among college students during the pandemic, while general well-being, physical activity^13^, and sleep quality^14^ decreased. First year and international students were the most strongly affected among the student community. Research in Japan shows that, due to having to adapt to an unfamiliar learning environment, academic distress was higher among first year students during the pandemic^15^.

While the negative impact of the pandemic on students’ mental health has been pointed out by researchers around the world, little is known about the mental health of faculty and staff. There is some evidence that mental health deteriorates more among students than university staff during the pandemic^16^. Odriozola-González and colleagues report that 60.31% of students and 45.07% of staff from the Arts and Humanities department of a Spanish university showed moderate to severe subjective distress during the outbreak of COVID-19 pandemic in May 2020^16^. The unexpected switch from face-to-face to online education placed a significant burden on the faculty, who had to redesign courses and assessment methods in a very short period of time, while dealing with all the other lifestyle changes imposed by COVID-19 restrictions. Research suggests that the way faculty dealt with the shift to online education affected not only their own mental health, but also students’ perceptions of the learning content. More specifically, faculty who perceived the shift as threatening experienced more burnout, and received more negative student evaluations^17^. As mental health issues among faculty can impact the quality of instruction, which may in turn affect students’ learning and psychological well-being, more research on faculty mental health during the COVID-19 induced emergency online education is necessary.

Even before the outbreak of the pandemic, poor mental health among academics has often been noted. Compared to other professions, university academic staff experience less job satisfaction and psychological health because of the high number of students^18^, heavy workload, long working hours, and lack of work–life balance^19,20^. These problems are even more pronounced in Japan, where faculty working time and the teaching load are higher than in other countries^21,22^. Most university academic staff in Japan report not having enough time for research because of long and frequent internal meetings, heavy teaching load, and administrative work^22^. The challenge brought by the unexpected shift to online education during the COVID-19 pandemic added to this already heavy workload. Japanese academic staff had no alternative but to spend extra time redesigning course content and learning to use new technologies for streaming and online meetings. However, to date, there is little empirical evidence on faculty mental health after the COVID-19-induced switch to online education in Japan.

### The current research

This study aims to examine the mental health of Japanese faculty members who taught online courses during the COVID-19 pandemic. The outbreak of COVID-19 forced all major Japanese universities in affected areas to switch to online learning in the spring of 2020. Prior to the start of the new academic semester in April 2020, the decision to switch to online education or to postpone the start of the new academic semester to May 2020 was implemented in universities across Japan^23–25^. However, the Japanese educational system was unprepared for this major change. A 2018 OECD study reveals that only 18% of Japanese lower secondary teachers reported letting students use IT for class projects frequently, compared to the OECD average of 53%^26^. Digitization of higher education also lags behind other developed nations^27,28^. One reason for this is related to the concern that IT use could have negative effects on students’ health and learning (e.g. leading to too much screen time)^28^. Nevertheless, the faculty had only one month until the start of the semester to redesign courses, acquire the necessary IT equipment and software, and adapt to remote work and frequent administrative changes. The urgency of having to prepare online classes in just about a month added to the stress experienced by faculty during this time. Face-to-face interactions with students and colleagues decreased abruptly, while workloads and stress increased. Furthermore, amid the rapid changes and conflicting administrative orders, support services from the university were insufficient or tardy^29^. Together, these factors took a heavy toll on the mental health of the faculty. To clarify the effects of COVID-19 prevention measures on mental health in higher education, we focus on faculty mental health during online education, to identify risk factors and develop prevention strategies. Bringing empirical evidence pertaining to the predictors of faculty mental health may open the way to the implementation of evidence-based practices to improve the work environment and the quality of higher education in post-COVID-19 Japan.

In this study, we focus on workload, use of IT, and university support as predictors of faculty mental health. First, in line with previous research suggesting that the teaching load and long working hours predict higher stress among academic staff^19,20^, we investigated the effect of the number of online courses taught, as well as the preparation time for these courses. We hypothesize that teaching a greater number of online courses, as well as spending more time on course preparation, negatively predicts faculty mental health during online education.

Second, one of the challenges of online education is dealing with technology, changing modes of assessment, and IT resources at home^30^. While tech-savvy faculty may easily make the transition from face-to-face to online education, dealing with new services and technologies necessary for video creation, content streaming, or file sharing, may place a considerable burden on those with little prior experience in using such applications. Accordingly, we hypothesize that the burden related to learning how to use IT negatively predicts faculty mental health during online education.

Third, considering the unexpected shift in instruction methods, support from the university is necessary to make this transition smoother. While some studies have highlighted the importance of workplace safety measures on employee psychological distress and performance during the pandemic^31^, very little is known about the effects of workplace support. When working remotely, faculty may be deprived of the usual social support from colleagues and administrative staff, and information sharing may be scarce. Providing support during the transition from face-to-face to online education may be a buffer against a decline in their mental health. Therefore, we hypothesize that satisfaction with university support services promotes better mental health among faculty members during online education.

## Results

### Demographical characteristics

Table 1 presents the demographic characteristics of the participants. The participants’ ages ranged from 20 to 70 s, and those in their 50 s were the largest age group (38.3%). The age distribution of participants approximately corresponded to the age distribution of the faculty members at the target university. Furthermore, this age distribution was almost the same as that of the national survey of Japanese universities^32^. More than half the faculty members have taught at universities for over 10 years. The faculty/department affiliations of the participants were as follows: commerce and management (12.9%), economics (10.1%), law (12.5%), social sciences (12.5%), language and society (4.9%), other (11.7%), and undisclosed (35.9%). After excluding the undisclosed category, there were no differences in proportion between affiliations (*χ*^2^ (5) = 10.9, *p* > 0.05). Faculty gave four online lectures on average every week during the COVID-19 pandemic. While they spent approximately 190 min to prepare a synchronous online course per class (per week), they devoted over 330 min to prepare an asynchronous online course. The average difficulty score for IT devices and the average satisfaction score for administrative support were 2.71 (standard error = 0.17) and 3.46 (0.22), respectively. Skewness and kurtosis of the difficulty score were − 0.31 and − 0.79, and those of the satisfaction score were − 0.57 and 0.05.

**Table 1:** Demographic characteristics.

Age group (%)	20–30 s	40 s	50 s	60–70 s
	17.7	26.6	38.3	17.3
Teaching experience (%)	< 5 years	5–10 years	10–20 years	> 20 years
	25.8	13.3	32.3	28.6
# of lectures as online education	4.22 ± 3.23 (1–20)^a^			
Preparation time per one class (min.) Synchronous online courses	192.0 ± 178.2 (0–1440)^a^			
Preparation time per one class (min.) Asynchronous online courses	331.9 ± 323.6 (7–2400)^a^			
Difficulty score for IT devices	2.71 ± 0.95 (1–4)^a^			
Satisfaction score for administration support	3.46 ± 0.87 (1–5)^a^			

^a^Average ± standard deviation (range).

### Associations among demographical characteristics

While the older generation did not spend much time preparing synchronous online courses (*r* = − 0.23, *p* < 0.01; Table 2), faculty members with longer teaching experience also did not devote much time to prepare for synchronous online courses (*r* = − 0.28, *p* < 0.001). These associations were not significant in the case of asynchronous online courses (*p* > 0.05). The difficulty score for IT devices was not significantly correlated with any of the variables except for the satisfaction score for administration support (*r* = − 0.24, *p* < 0.001).

**﻿Table 2:** Associations among demographic characteristics.

	Age	Teaching experience	# of lectures	Preparation time synchronous	Preparation time asynchronous	Difficulty score for IT devices
Teaching experience	0.53***					
# of lectures	0.07	0.31***				
Preparation time synchronous	− 0.23**	− 0.28***	− 0.18**			
Preparation time asynchronous	− 0.14	− 0.12	− 0.08	NA^a^		
Difficulty score for IT devices	0.09	0.05	− 0.03	0.04	0.02	
Satisfaction score for admin. support	0.06	− 0.02	− 0.06	− 0.04	− 0.17	− 0.24***

***: *p* < 0.001, **: *p* < 0.01, *: *p* < 0.05.

^a^Not available: correlation was not computed because some participants provided either a synchronous or asynchronous online course.

### Mental health before and during the COVID-19 pandemic

**Figure 1:**
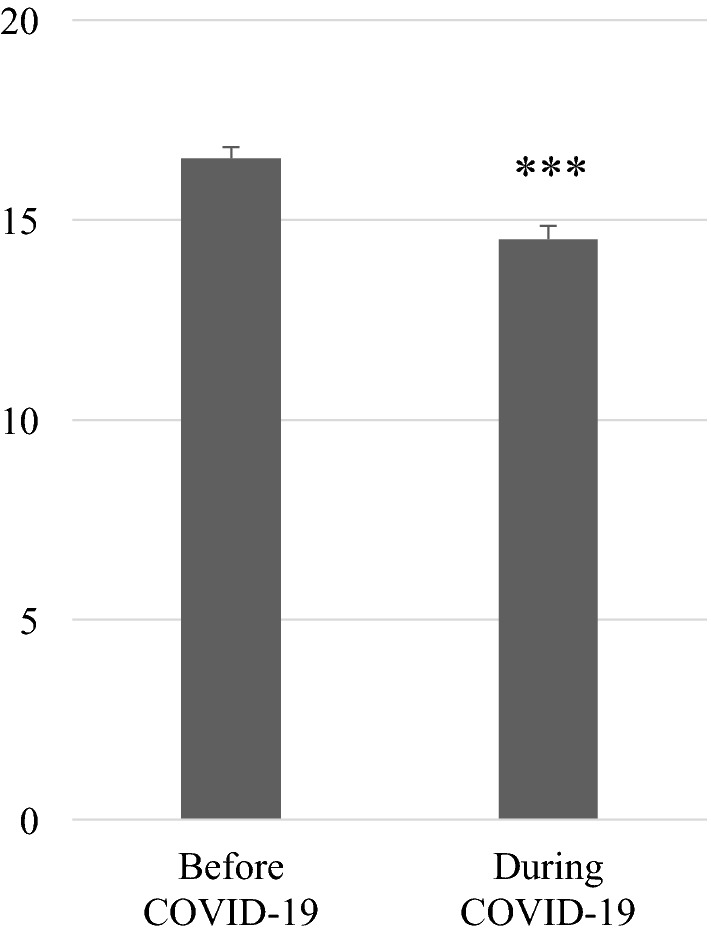
WHO-5 score before and during the COVID-19 pandemic. *bar* average, *error bar* standard error. ***: *p* < .001.

The WHO-5 score during the COVID-19 pandemic (mean (standard error) = 14.5(0.9)) was significantly lower than before (mean = 16.5(1.1); *t*(247) = 8.59, *p* < 0.001), thereby showing that the mental health of faculty members worsened during the COVID-19 pandemic (Fig. 1). We also found that 15.3% (i.e., 38 participants) of the faculty members were at risk before the pandemic and 33.5% (i.e., 82 participants) were at risk during the COVID-19 pandemic.

### Risk factors for mental illness

A binomial logistic regression analysis revealed two significant risk factors for mental illness among faculty members (Table 3), while the validity of the regression model was confirmed by the Hosmer–Lemeshow test (*χ*^2^(8) = 2.92, *p* = 0.94). The first risk factor was the difficulty score for IT devices (*β* = 0.35, odds ratio = 1.42, *p* = 0.024), and the faculty members who were not good at using IT devices were more susceptible to mental illness. Another was the satisfaction score for administrative support (*β* = − 0.49, odds ratio = 0.61, *p* = 0.003), and faculty members who were satisfied with the administrative support maintained good mental health during the COVID-19 pandemic. We did not find any significant predictive variables, such as age group, teaching experience, number of lectures, and preparation time (*ps* > 0.05).

**Table 3:** Results of a binomial logistic regression analysis for mental illness.

	*β*	SE	OR	95% CI	*p* value
Difficulty score for IT devices	0.35	0.16	1.42	1.05–1.93	0.024
Satisfaction score for administrative support	− 0.49	0.16	0.61	0.45–0.85	0.003

*β* standardized partial regression coefficient, *SE* standard error, *OR* odds ratio, *95%CI* confidence interval.

## Discussion

The working environment of university faculty changed rapidly during the COVID-19 pandemic. Faculty members were asked to switch from in-person instruction to teaching classes online in a very short period of time, as part of efforts to stop the spread of the COVID-19 pandemic^15^. Against this backdrop, this study investigated the mental health of Japanese faculty members who taught classes online during the COVID-19 pandemic, to identify risk factors for poor mental health and prevent the development of mental illness in the future. Although other studies have examined the mental health of university students during the COVID-19 pandemic^3,10–12^, relatively few studies have focused on the mental health of faculty members in universities. Accordingly, our study contributes to the literature by providing new findings on the topic.

First, we investigated the actual condition of the faculty’s mental health before the COVID-19 pandemic. Even before the outbreak of the pandemic, it had been reported that faculty members in universities have poor mental health compared to members of other professions^18^. We used the WHO-5 to measure the mental health of faculty members and then calculated the proportion of faculty at risk of mental illness (total WHO-5 score < 13). The results revealed that 15.3% of faculty members had been at risk of developing a mental illness, even before the COVID-19 pandemic. Another investigation of mental health among Japanese faculty reports that 10.2% of faculty members were at risk for mental illness prior to the pandemic^33^. Compared to this result, the at-risk group was larger in our sample. Lee et al.^34^ also used the WHO-5 to assess the mental health risks of various occupations. They reported that 13.2% of management/professionals were at risk of developing mental illnesses. In the context of their findings, the proportion of faculty members at the risk of developing a mental illness is comparatively high, thus demonstrating that the mental health of faculty members in universities is inherently worse than that of workers in the management/professional field. Lee et al.^34^ also reported that the proportion of office workers at the risk of mental illness was 12.9%. Thus, the proportion of faculty members at the risk of developing mental illness exceeded that of office workers. It is quantitatively evident that the mental health of faculty members in universities had been worse than that of workers in other occupations, even before the COVID-19 pandemic.

Next, we focused on the WHO-5 scores of faculty members before and during the COVID-19 pandemic, which revealed that the mental health of faculty members worsened during the pandemic. The proportion at risk of mental illness was 15.3% before the COVID-19 pandemic, but nearly doubled to 33.5% during that period. We speculated that this large increase was due to lifestyle and work-related changes, including remote work, a lack of face-to-face communication, and the shift to online instruction in a very short period of time. In particular, the sudden transition to teaching classes online involved a very heavy workload, accompanied by unforeseen financial and time costs^35^.

In addition, we hypothesized that the dramatic decline observed in the mental health of many faculty members could be attributed to four risk factors: the number of classes taught online, the time needed to prepare for those classes, challenges related to the technology needed to conduct classes online, and the level of satisfaction with support services provided by the university. Our results suggest that two of these were significant risk factors for the poor mental health among faculty members. The first risk factor was related to technology. Faculty members who reported having difficulty using the required technology were more susceptible to poorer mental health. The second risk factor was the level of satisfaction with the university support services. Faculty members who reported higher levels of satisfaction with university support services maintained good mental health despite the unforeseen shift in the mode of instruction. When faculty members first began teaching their classes online, many of them were not familiar with the online conferencing software, lacked the required equipment (e.g., webcams, high-quality microphones), and received limited, if any, training on online content delivery^36^. Furthermore, the lack of relevant IT skills and experience made it difficult for these individuals to adapt to teaching classes online^17^. Faculty members who lacked IT skills had to redesign their courses and learn IT skills simultaneously. In this situation, it is speculated that faculty members who had difficulty in using IT felt a substantial burden and decline in their mental health.

In addition, the results revealed that the amount of satisfaction with university support services for online teaching was related to good mental health. To reduce difficulty in using IT, it is important to ensure that the working environment of the faculty satisfies the needs of the faculty who must use unfamiliar technology to teach classes online^37^. According to Wang and Li^37^, the needs of the faculty broadly refer to the support that universities must provide for faculty members to effectively use new technology (organizational level) and the technology that helps them meet the objectives of their job (technological level). It also involves assistance from their colleagues, which helps them effectively use technology at work (people level). The administrative support services for online teaching satisfied all the requirements listed above. For example, the university provided social support such as consultations with university IT staff, who explained how to use the software and equipment needed for online instruction, as well as technical support such as providing equipment and writing manuals for some software. Satisfaction with this comprehensive support provided by the university might have reduced the faculty members’ difficulty in using IT, and consequently, improved their mental health.

Our results also showed that both the number of classes taught online and class preparation time were weak predictors of mental health among faculty during the COVID-19 pandemic, as compared to challenges related to the technology needed to conduct classes online. This result suggests that the psychological burden of dealing with unfamiliar technology, rather than the workload resulting from online classes, including the long preparation time, had a substantially negative effect on the mental health of faculty members.

The workload for faculty members can be broadly divided into three categories: teaching, research, and service. Faculty members are required to strike an appropriate balance between the three. According to Zey-Ferrell and Baker^38^, faculty members recognize that teaching is the main component of their work. Their study investigated 503 faculty members, and found that although 92.1% had strong expectations from themselves about teaching, such ideal self-expectations were incongruent with what they actually did. Furthermore, there are a few serious stressors for faculty members, including heavy workloads and anxiety related to securing funding for their research, but the most serious was excessively high self-expectations^39,40^. Taking these findings into consideration, it is possible that during the COVID-19 pandemic, faculty members placed high expectations on themselves, aiming to provide high-quality lessons online and had to simultaneously deal with the unfamiliar technology needed to conduct classes online. Such circumstances can be reasonably expected to cause stress, which leads to poor mental health.

In Japan, some university classes were held in person after the lockdown was lifted. However, many courses continue to be conducted online. Some faculty members consider the shift to online teaching to be a positive challenge or at least useful for developing certain competencies^17^. A previous study also revealed that online classes can be useful, effective, and have a positive influence on student performance^41^. Furthermore, with online classes, faculty members and students do not need to spend time and money to commute, and there is less drain on university resources. This leads to benefits such as conserving the time and energy of the faculty and saving university resources^42^. Based on these findings, we assume that online classes will become a normal part of university education, and that faculty members will therefore continue to teach classes online to some extent. Accordingly, universities will need to provide both technical and social support to reduce faculty members’ difficulty in using IT and maintain their mental health.

We established the effect of teaching classes online during the COVID-19 pandemic on the mental health of faculty members in universities, but there were some limitations to our research, related to sampling and measurement. As sampling issue, we first acknowledge that the number of participants in our study was quite limited, and included only Japanese faculty members. The extent of the COVID-19 infection and government countermeasures differ across countries. In addition, the utilization of online services to deliver course instructions in the setting of higher education varied according to country, before the COVID-19 pandemic. Therefore, the results may not be generalizable to other countries. Furthermore, depending on the major (e.g., medical science and nursing science), some practical subjects may have been more difficult than others to adapt to online instruction. This study investigated a Japanese university specializing in social science; therefore, the results may not be generalizable to other institutions of higher education. Accordingly, we need to widen the scope of participants to include faculty members from various departments and institutions in more countries in future research. Finally, due to missing data, we could not investigate gender differences. The switch to online education and remote work may have affected women and men differently. For example, previous research suggests that during COVID-19, women carried a heavier load in the provision of childcare^43^. Therefore, future research should look deeper into gender differences in mental health among academic staff during the pandemic.

As for measurement issues, mental health before the pandemic was reported retrospectively, so memory biases could have affected participants’ evaluations, rendering them unreliable. Even so, retrospectively evaluated average well-being in our study was similar to that reported in previous studies employing the Japanese version of WHO-5^44^, therefore retrospection might not have critically affected participants’ evaluations. In addition, because we measured difficulty in using IT devices and satisfaction with university support services with one item each, our results should be interpreted with caution. To provide a more detailed image of the problems causing poor mental health among faculty teaching online, validated scales measuring different aspects of university support (e.g. technical vs social support) and IT difficulty (e.g. lack of expertise in using IT vs stress produced by technical problems, etc.), alongside longitudinal assessments of well-being should be used in future research.

Our research focused on the first year of the COVID-19 pandemic, during which most faculty members in universities were required to shift to teaching their classes online. Accordingly, these faculty members had to adapt their lessons for online instruction in a very short period of time. In fact, many faculty members were required to set up equipment and learn the necessary IT skills, and in many cases, redesign the content of their lessons in just a month. Accordingly, they might have felt overloaded. More than a year after the outbreak, the work of adapting lessons for online instruction is mostly complete, and thus, the burden on the faculty may be less severe in the future. This change might ultimately have a positive effect on the mental health of faculty members. Regardless, the results of this study demonstrate the need to continuously monitor the mental health of faculty members who must teach classes online in universities.

This study has focused on the mental health of university faculty, but our findings may possibly be applicable to other occupations as well. The COVID-19 pandemic has been found to cause psychological stress for people working in various occupations, with new work-styles such as telework and remote work being identified as the primary cause of such stress^45^. In addition, it has been shown that during the COVID-19 pandemic utilizing IT has become more important and the need to use IT has become more frequent in comparison to pre-pandemic times^46^. This situation of work-styles changing due to the pandemic and mental health worsening due to increased use of IT may be viewed as similar to the situation experienced by university faculty. Therefore, the findings of this study may possibly be applied to other occupations as well, in order to explain the cause of the deterioration of mental health from the perspective of degree of familiarity with IT use and satisfaction with company support, thus clarifying the kind of support that companies must offer to promote the continuation of telework.

## Conclusions

We investigated the mental health of faculty members who were required to suddenly shift to online instruction because of the COVID-19 pandemic. We found that the mental health of faculty members was worse during the pandemic than before. The results showed that 33% of faculty members were at risk of mental illness during the pandemic. We attempted to clarify the risk factors involved in the dramatic decline in mental health among university faculty. We found that university support services, consisting of both technical and social support, had a positive effect on the mental health of faculty members, whereas the stress caused from using unfamiliar technology had a negative effect. If online instruction continues in the post-COVID-19 era, universities should make efforts to maintain the mental health of faculty members by continuously providing adequate support.

## Methods

### Participants

We recruited participants from a Japanese university with 537 faculty members who gave lectures during the COVID-19 pandemic (April 2020 to February 2021). The university was selected because it is located in Tokyo, where the situation was the worst in Japan, and almost all lectures were swiftly switched to online modes, such as synchronous (e.g., real-time online learning) and asynchronous (e.g., video learning) online courses. Faculty members were required to prepare and provide lectures online, even though they did not have much experience and expertise in online education. The university provided them with administrative support, such as consultations with its IT staff, who explained how to use the necessary software and equipment, as well as technical support in the form of equipment and writing manuals for software. The questionnaire was delivered to faculty members via an in-house online network or paper style. We collected 248 questionnaires from faculty members (response rate = 46.2%). Prior to their engagement in the study, we briefed the faculty members about the study, and obtained informed consent from them. The research protocol was approved by the ethics committee of Hitotsubashi University (approval number 2020C012; Tokyo, Japan) and all methods were carried out in accordance with the Declaration of Helsinki.

### Measures

#### Demographic characteristics and online education features

In addition to the age and teaching experiences of faculty members, several characteristics of online education were also measured. The number of lectures provided as online education was counted for each faculty member, while preparation time per class was evaluated depending on either synchronous or asynchronous style. To measure faculty’s difficulty in using IT devices, we created one item (“How much trouble did you have operating information technology (IT) devices such as computers, network devices, and apps?”), measured on a 4-point Likert scale (1 = not problematic; 4 = very problematic). We also assessed faculty’s satisfaction with the administrative support from the university using one item (“How satisfied were you with the support provided by the university for your online courses?”) measured on a 5-point Likert scale (1 = unsatisfied; 5 = satisfied), because they were provided several resources and materials for their lectures by the administration. For example, the following support services were included: distribution of instruction manuals for online education, free seminars on online education, free video conference account, free rental of studios to record/edit lectures, free rental of IT gadgets (e.g., web cams; high-quality microphones), technical support consultations for online education, consultation with experts on lecture materials (e.g., copyright).

#### Mental Health

The mental health of faculty members was assessed using the Japanese version of the World Health Organization-Five Well-Being Index (WHO-5)^44^. The WHO-5 was originally introduced by the World Health Organization in 1998^47^, and has high reliability and validity in several countries, including Japan. The WHO-5 is a 5-item questionnaire, and each item is measured on a 6-point Likert scale ranging from 0 (“at no time”) to 5 (“all of the time”). The total score is calculated by the sum of the item scores (i.e., 0–25). Lower total scores reflect worse imaginable well-being. Mental health during COVID-19 was assessed using the WHO-5, while mental health before the pandemic was retrospectively evaluated in the same way, for example: “Did you feel cheerful and in good spirits in 2019?”. In line with previous studies^44^, we set cut-off values of total score < 13, thus showing that the participants were at risk for mental illnesses such as major depression.

### Data analyses

First, the descriptive statistics for the characteristics of online education were computed. Second, we calculated Pearson’s correlation coefficients among these variables to reveal several associations, such as preparation time (i.e., working hours) and difficulty score for IT devices. Third, we investigated whether the mental health of the faculty members worsened during the COVID-19 pandemic, using a paired samples t-test on WHO-5. There were no outliers (scores five standard deviations either below or above the mean). The number of faculty members at risk of mental illness was also counted based on the cut-off values of WHO-5. Finally, a binomial logistic regression analysis was conducted to examine the risk factors for mental illness among faculty members during the COVID-19 pandemic. Demographic characteristics and features of online education were used as predictive variables (i.e., age group, teaching experience, number of lectures, preparation time, difficulty score for IT devices, satisfaction score for administration support), and the presence or absence of mental illness (i.e., < 13 or above 13) was used as a dependent variable. A step-wise procedure was introduced with the inclusion criterion *α* < 0.05, and the non-significant variables were excluded based on this criterion. While we computed standardized coefficients (*β*) and odds ratios with 95% confidence intervals, the Hosmer–Lemeshow test was performed to evaluate the goodness of fit for our logistic regression model. Statistical analyses were conducted using R version 3.3.3^48^.
